# Research Progress in Coconut Water: A Review of Nutritional Composition, Biological Activities, and Novel Processing Technologies

**DOI:** 10.3390/foods14091503

**Published:** 2025-04-25

**Authors:** Shaoran Shi, Wenxin Wang, Fengzhang Wang, Peiqing Yang, Huanzhi Yang, Xiyu He, Xiaojun Liao

**Affiliations:** 1 College of Food Science and Nutritional Engineering, China Agricultural University, Beijing 100083, China; 2 Institute of Food Science and Technology, Chinese Academy of Agricultural Sciences, Beijing 100193, China; 3 College of Mechanical and Electrical Engineering, Hunan Agricultural University, Changsha 410128, China

**Keywords:** coconut water, nutritional composition, biological activities, novel processing technologies

## Abstract

Coconut (*Cocos nucifera* L.) is a nutrient-rich plant extensively cultivated in tropical and subtropical regions. Coconut water (CW), the primary edible component of the fruit, has gained significant attention due to its nutritional value and increasing popularity as a functional beverage. In addition to its hydrating properties, CW is rich in essential nutrients such as sugars, minerals, and vitamins, which contribute to its diverse biological activities, including antioxidant, anti-inflammatory, anti-cancer, cardioprotective, and antimicrobial effects. However, CW’s high perishability and susceptibility to rapid deterioration present significant challenges for its preservation. The growing demand for natural and fresh CW has driven the development of innovative technologies aiming at extending its shelf life while maintaining its nutritional quality and sensory attributes. This review highlights recent research advancements in CW, focusing on its nutritional composition, biological activities, and innovations in preservation technologies. The aim is to facilitate the optimization of CW beverage formulations, promote the adoption of effective preservation methods, and drive the development of high-quality and consumer-appealing CW products.

## 1. Introduction

Coconut (*Cocos nucifera* L.) is a tropical fruit from the areca family, widely distributed in regions such as West and East Africa, Southeast Asia, the Pacific islands, as well as the Americas [1,2,3]. Globally, coconut cultivation covers 12.3 million hectares, yielding 61.4 million tons annually. The main producers include Indonesia, the Philippines, India, Brazil, and Sri Lanka, where coconut palm grows naturally [4]. Although the precise number of coconut tree species remains uncertain, three primary groups have emerged through both natural and artificial selection: the tall (or typical) variety, the dwarf (or nana) variety, and hybrid varieties [2]. Tall coconut species are often cultivated for oil production, whereas dwarf species are favored for the production of fresh coconut water (CW) and use in desserts. Hybrid coconut species combine the advantageous traits of both tall and dwarf coconut cultivars, meaning they exhibit a greater nut yield, higher oil extraction rates, and markedly improved pest/disease resistance versus conventional varieties, making them superior for commercial cultivation [2,5]. Coconut cultivation serves as a crucial strategy for enhancing food security in tropical regions, providing essential nutrients such as protein and fat, as well as a sustainable source of energy, while simultaneously generating valuable employment opportunities [6].

A mature coconut fruit consists of approximately 51.7% kernel, 38.5% shell, and 9.8% water. The kernel, which is the solid endosperm, is enclosed within a hollow shell that contains CW, alternatively referred to as the liquid endosperm. CW constitutes approximately 25% of the total weight of the fruit [7]. Tender coconut water (TCW) refers to the clear, slightly sweet, and aqueous component of an immature coconut [8]. It contains about 95.5% water, 0.1% fat, 4% carbohydrate, 0.02% calcium, 0.01% phosphorus, 0.5% iron, and some amino acids, vitamin B complex, vitamin C, and mineral salts [9]. TCW is rich in natural phytonutrients and has gained popularity as a natural energy drink [10]. Furthermore, TCW can be preserved for a duration of up to 15 days in ambient conditions after being harvested from trees. As the coconut fruit matures, the volume of CW decreases, and its chemical composition undergoes changes: the total soluble solids (TSS), ash content, and mineral content decrease, whereas the fat and protein content increase.

CW possesses various functional properties, including antioxidant [11], antimicrobial [12], antidiabetic [13], anti-inflammatory [14], anti-hyperlipidemic, anti-cancer [15], anti-ulcerogenic, and cardioprotective effects [16]. It has been incorporated into traditional medicine and dietary interventions in numerous cultures, particularly in Africa, India, and the Philippines, for the treatment of a multitude of conditions, including gastroenteritis, coronary heart disease, kidney diseases such as urolithiasis, and urinary tract infections. As a result of its nutritious and functional attributes, CW has gained popularity as a natural beverage, attracting increasing consumer interest [17]. CW has solidified its status as a premium functional beverage, with surging global demand propelling it to become one of the most rapidly expanding categories in the plant-based drinks sector [2,18].

Although CW is a popular natural functional beverage, it is highly susceptible to deterioration due to microbial contamination and enzymatic activity once exposed to the external environment [8]. This susceptibility drastically shortens its shelf life and can lead to substantial changes in its biochemical composition and organoleptic properties, resulting in the loss of nutritional values [19]. Therefore, it is imperative to utilize food processing technologies to ensure the preservation of CW and the successful commercialization of its related products. Over the past few decades, the pursuit of pioneering food processing technologies has intensified, with a focus on techniques such as high-hydrostatic-pressure processing, high-pressure carbon dioxide treatment, cold plasma application, pulsed electric fields, pulsed light technology, UV-C irradiation, ultrasonic processing, ozone treatment, and microfiltration. These innovative approaches are meticulously designed to enhance the preservation of both the nutritional integrity and sensory qualities of food products.

**Figure 1:**
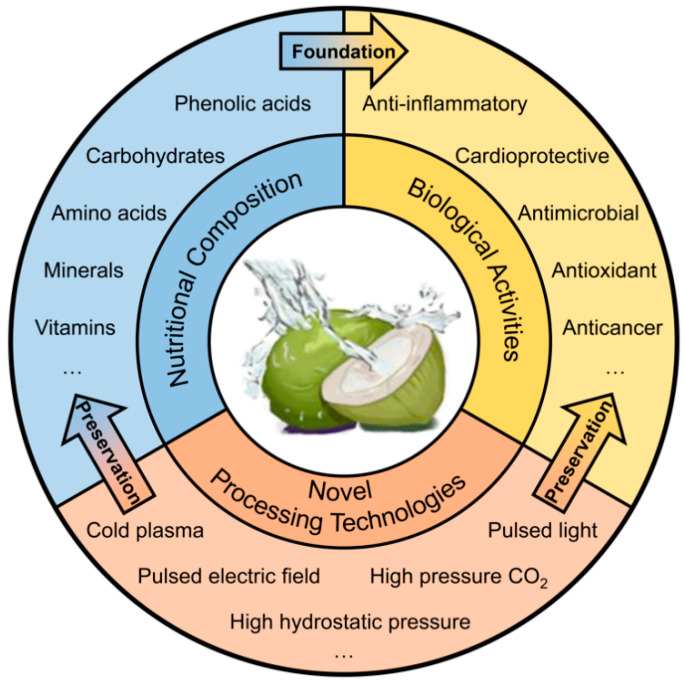
Overview of the nutritional composition, biological activities, and novel processing technologies on coconut water.

In summary, the coconut stands as a multifaceted fruit, boasting remarkable nutritional benefits and a plethora of natural nutrients. To the best of our knowledge, no recent reviews have systematically summarized the diverse aspects of coconut water (CW). This article provides a comprehensive overview of CW, emphasizing its nutritional constituents, bioactive properties, and the latest progress in processing technologies (Figure 1). The insights garnered are intended to steer the advancement, intricate processing, and enhanced value creation within the CW industry.

## 2. Nutritional Composition of CW

**Figure 2:**
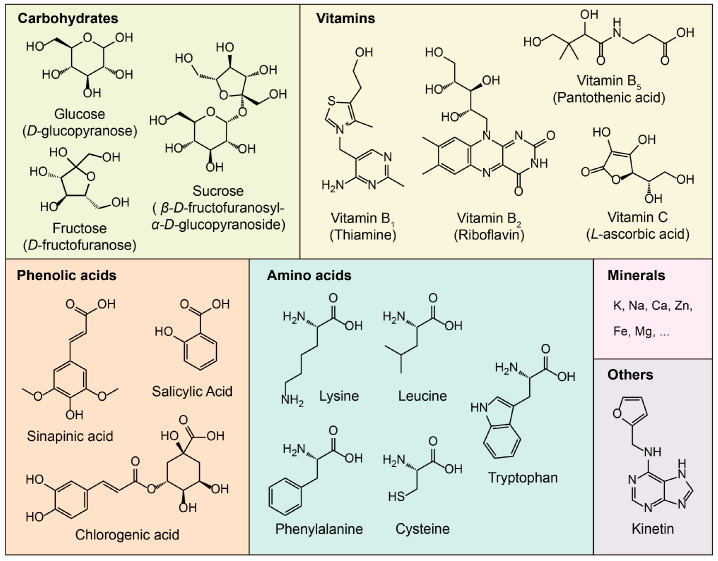
Primary nutritional compounds of coconut water.

CW is widely recognized as a functional beverage due to its rich and balanced nutritional composition. It provides a well-balanced blend of various compounds, such as carbohydrates, vital minerals (such as potassium (K), calcium (Ca), and magnesium (Mg)), B-complex vitamins and vitamin C, phenolic acids, and amino acids (Figure 2). These components not only contribute to its hydrating properties but also enhance its potential health benefits, making CW a naturally refreshing and nutritionally valuable drink.

### 2.1. Carbohydrates

The carbohydrates in CW are predominantly composed of natural sugars, with glucose, fructose, and sucrose being the dominant types [20]. These sugars serve as an immediate and accessible source of energy, with their content varying depending on the coconut variety and its stage of maturity. According to USDA data in 2018, one hundred milliliters of pure CW contain 3.39 g of carbohydrates, including 1.27 g of sugars. TCW, obtained from immature coconuts aged between 5 and 7 months, exhibits TSS ranging from 3.8 to 6.9 °Bx, with a total sugar content varying from 2.08 to 6.52% [20]. The levels of sugar and TSS fluctuate during maturation, initially increasing and then decreasing [10]. During the initial stages, specifically 4 to 5 months post-pollination, CW exhibits a sour and astringent taste profile. At 7–8 months, CW acquires a sweet flavor profile, accompanied by an increase in sugar content to approximately 5 to 6% [21]. A typical 100 mL sample of TCW comprises approximately 95% water, 5.5–7% non-reducing sugars, and 4–4.5% reducing sugars. Due to its relatively high sugar content, TCW is prone to microbial contamination, necessitating processing methods to eliminate pathogens and prolong shelf life [22]. The total sugar content in mature CW varies depending on cultivar and geographic location, typically ranging between 1.8 and 4.4 g/100 mL. In comparison, mature CW contains 0.2% reducing sugar, while TCW has a higher sugar content of 4.4% [23].

### 2.2. Minerals

CW is highly valued for its rich mineral content, which is essential for maintaining hydration and supporting various bodily functions. The primary minerals present in CW include K, sodium (Na), Ca, Mg, zinc (Zn), and iron (Fe), with K being the most prevalent among them [10]. A 100 mL serving of TCW typically consists of 95% water and notable mineral concentrations, such as K (290 mg), Fe (106 mg), Ca (44 mg), Na (42 mg), and copper (Cu, 26 mg) [22]. In another work, fresh TCW, a naturally sterile liquid harvested from immature coconuts aged 5 to 7 months, showed the following mineral composition: Na (1.75–31.4 mg/100 g), K (203.7–249.0 mg/100 g), Ca (3.6–27.35 mg/100 g), Mg (6.4–25.0 mg/100 g), and Fe (0.02–4.2 mg/100 g) [20]. After an 8-month period, the flavor intensity of CW diminishes, whereas its mineral content increases. Furthermore, the mineral and nutrient compositions of CW can vary depending on regional factors [21].

### 2.3. Vitamins

Vitamins are essential for various physiological functions in the human body. CW contains several water-soluble B-complex vitamins, including thiamine (B1), riboflavin (B2), pantothenic acid (B5), pyridoxine (B6), biotin (B7), and folate (B9). In addition to these B vitamins, CW also contains vitamin C, a key dietary antioxidant that supports immune function and aids in tissue repair [24]. These vitamins play crucial roles in energy production, metabolism, and maintaining overall health [24].

### 2.4. Phenolic Compounds

The composition of phenolic catechins in CW has scarcely been reported, with salicylic acid identified as the predominant phenolic compound. A comprehensive GC-MS analysis has revealed the presence of 19 water metabolites, encompassing phenols, within CW [10]. Among the 41 compounds identified, the major phenolic compounds include chlorogenic acid, caffeic acids, epicatechin, *L*-epicatechin, proanthocyanidin B1 and B2, catechin, tocopherols, and salicylic acid [25,26]. Additionally, the content of phenolic compounds in CW increases with maturity, enhancing its antioxidant properties and nutritional value [27].

### 2.5. Amino Acids

Amino acids are essential for various metabolic pathways crucial to life [28]. CW contains abundant free amino acids, highlighting its potential as a valuable nutritional resource, including essential ones such as lysine, leucine, cysteine, phenylalanine, histidine, and tryptophan [29]. Yannam et al. found that CW contains nine essential amino acids, with tryptophan being the most abundant [18]. Halim et al. used high-performance liquid chromatography (HPLC) to demonstrate that the total amino acid content increases as the coconut matures [28]. The primary amino acids identified were glutamine, alanine, and tyrosine [28]. Interestingly, CW contains higher levels of alanine, arginine, cysteine, and serine compared to milk, and these amino acids might be associated with the discoloration of CW [30]. TCW, in particular, is rich in arginine, alanine, cysteine, and serine [31]. Among these, arginine plays a significant role in producing nitric oxide, which enhances blood flow, promotes vasodilation, and boosts endurance [28]. Furthermore, L-arginine supports growth hormone secretion, aids muscle protein synthesis, and has been found to transform pancreatic cells into insulin-producing cells, potentially reversing diabetes-related effects [32].

### 2.6. Other Compounds

In addition to sugars (e.g., glucose, fructose, and sucrose), minerals, vitamins, phenolic acids, and amino acids, CW also contains certain amounts of phytohormones, particularly cytokinin, as well as other compounds like lipids, nitrogenous compounds, and enzymes [29]. Furthermore, CW has been found to contain a variety of volatile aromatic compounds, including esters, alcohols, aldehydes, phenols, and ketone, with ester compounds contributing the most to its aroma profile [25].

## 3. Biological Activities of Coconut Water

CW is a refreshing and rehydrating beverage rich in vitamins, minerals, electrolytes, amino acids, growth-promoting factors, and proteins [33]. It is free from fat and low in calories. CW possesses various therapeutic properties as listed in Table 1, with various applications ranging from foods to cosmetics. Numerous scientific studies have reported antioxidant activities in tender coconut water, but the comprehensive information on other biological activities of CW is scare. Therefore, the bioactivities of CW are reviewed in this article to provide an integrated understanding.

### 3.1. Antioxidant Activity

The aromatic dwarf variety of CW has been reported to contain various phenolic compounds. Key bioactive constituents include catechin and several phenolic acids, such as syringic acid, salicylic acid, *m*-coumaric acid, *p*-coumaric acid, and *p*-hydroxybenzoic acid, which serve as significant sources of antioxidants in the prevention of diseases [43]. Caffeic acid, identified in CW from the Malayan Green Dwarf variety, has been reported to contribute to its antioxidant activities [44]. CW from young Malayan Yellow Dwarf exhibited antioxidant and anti-aging effects, with its rich bioactive composition holding promise as a natural functional ingredient for anti-aging skincare, underscoring its cosmetic industry potential [28]. The catechin extracted from CW showed antioxidant, antibacterial, and anti-cancer effects [45]. Interestingly, green CW exhibits a stronger free radical scavenging activity, higher antioxidant and anti-aging activities, and a richer profile of bioactive compounds compared to mature CW [28,46]. Furthermore, the DPPH and ABTS radical scavenging activities of CW were found to be comparable to or even higher than those of ascorbic acid, while its anti-collagenase activity surpassed that of epigallocatechin gallate (EGCG) [28].

In addition, CW also contains a group of phytohormones, namely cytokinins such as kinetin, kinetin riboside, trans-zeatin, trans-zeatin riboside, and trans-zeatin glucoside [47,48]. A previous study showed that kinetin and zeatin are used in cosmetic products due to their antioxidant and anti-aging properties [24]. Moreover, the essential oil extracted from CW, primarily comprising esters (58.3%) and ketones (33.5%), has also demonstrated notable free radical scavenging activity and antioxidant potential [49]. An in vivo study on Wistar albino rats confirmed that CW extract can enhance antioxidant capacity, highlighting its potential health benefits [34].

Interestingly, a recent study also showed that CW could inhibit the browning of ‘Gala’ apple wedges during storage at 4 ± 1 °C for 9 days, suggesting its promising application as a natural anti-browning agent in fresh-cut products [50]. Therefore, the richness of beneficial bioactive compounds in CW makes it a potent natural source of antioxidants and anti-aging compounds.

### 3.2. Anti-Inflammatory Activity

Rao et al. explored the anti-inflammatory activity of CW of different maturation stages (young and mature) with a rat paw edema model (4 mL/100 g dose orally) of inflammation using plethysmometer [14]. The results revealed that the maximum percentage inhibition observed was 42.52% for young CW and 25.94% for mature CW, respectively. Notably, the anti-inflammatory effect of young CW was significantly greater than that of orally administered ibuprofen at a dose of 400 mg/70 kg. These findings strongly indicate the potential use of young CW for a potent anti-inflammatory effect and mature CW for a moderate anti-inflammatory effect. Furthermore, the effect of TCW on primary rat hepatocyte viability, cytokine-induced gene expression, and proinflammatory signaling in an in vitro model of sepsis was also verified. TCW could represses hepatocyte IL-1β-mediated inflammatory damage by inhibiting Nos2 mRNA and iNOS protein expression through the AKT and JNK signaling pathways in vitro, with decreasing hepatocyte expression of pro-inflammatory cytokines and increased expression of acute-phase proteins Serpine1 and HMOX1, demonstrating that TCW could potentially be beneficial as a therapeutic agent in conditions where hepatic Nos2 expression is upregulated [35].

### 3.3. Ant-Proliferative Activity

Anionic host defense peptides (AHDPs) are gaining attention as key elements of the innate immune system and as potential antimicrobial agents with unique modes of action. Cn-AMP2 (TESYFVFSVGM), an AHDP from green CW of the plant Cocos nucifera, showed anti-proliferative activity against the human glioma cell lines 1321N1 and U87MG, with IC50 values of 1.25 and 1.85 mM, respectively [15].

### 3.4. Cardioprotective Protect

The richness of macro- and micro-nutrients in TCW is reported to have hypolipidemic, cardioprotective, and hepatoprotective effects [51]. The therapeutic properties of TCW make it useful as a remedy for a lot of ailments. Male Sprague–Dawley rats fed with a fructose-rich diet and treated with TCW (4 mL/100 g of body weight) for 3 subsequent weeks showed a significantly lowered systolic blood pressure, serum triglycerides, and free fatty acids. Plasma glucose and insulin levels and lipid peroxidation markers such as MDA, hydroperoxides, and conjugated dienes were also significantly reduced [11]. Oral administration of mature CW in diabetic rats showed a significant reduction in blood glucose and glycated hemoglobin levels, with an improvement in plasma insulin levels, which exerted significant antihyperglycemic potential and could be developed as a potent drug candidate or nutraceutical for the management of diabetes and associated complications [32]. Furthermore, both chronic oral administration of TCW (4 mL/100 g/day for 30 days) and acute intravenous treatment with lyophilized TCW (250 mg/100 g, single dose) significantly attenuated isoproterenol-induced oxidative stress and demonstrated notable antithrombotic effects [36]. When compared to streptokinase, one of the most effective thrombolytic drugs, TCW demonstrated a superior antioxidant activity and comparable antithrombotic efficacy. Similarly, oral MCW treatment (4 mL/100 g/day, 45 days) significantly lowered both blood glucose and HbA1c levels in diabetic rats while displaying dual antidiabetic and antithrombotic effects, likely via the L-arginine-NO pathway [13].

TCW treatment (oral: 4 mL/100 g/day for 30 days; 250 mg/100 g single dose) significantly reduced ISO-mediated oxidative stress and exhibited marked antithrombotic activity. Recent studies with TCW indicated that it is a rich source of cardioprotective factors viz. *L*-arginine [52], magnesium, potassium, calcium, and vitamin C, which are known to reduce the risk of coronary heart disease.

### 3.5. Antimicrobial Activity

The biosafety of CW and coconut oil was investigated by assessing their effects on the types and population of bacterial flora in the ileum of apparently healthy Wistar (AHW) rats. After four weeks of daily orogastric administration of various volumes of CW (0.5 mL, 1.0 mL, 1.5 mL, and 2.0 mL, respectively), it was observed that the CW exhibited inhibitory effects on the growth of all tested bacteria (*Escherichia coli*, *Proteus mirabilis*, *Pseudomonas aeruginosa*, *Klebsiella pneumoniae*, *Shigella flexneri*, *Serratia marscense*, and *Morganella morganii*) isolated from the ileum of the rats with diameter zones of inhibition ranging from 7.50 ± 0.50 mm to 23.00 ± 2.00 mm [37]. In the in vivo assay, CW reduced the population of the ileal bacterial flora of the rats, with the highest effect on Escherichia coli from 2.39 × 10 to 1.23 × 10 cfu/mL after 28 days of administration [37].

An in vitro experimental study was conducted to evaluate the antimicrobial efficacy of TCW in its natural state on Streptococcus mutans [12]. However, neither fresh nor pasteurized TCW exhibited antibacterial activity, whereas chlorhexidine, used as a positive control, demonstrated effective *S. mutans* inactivation. Moreover, natural bioactive antimicrobial peptides have been found from green coconut water [53]. CW is antiseptic and acts as a mouth cleanser. The antibacterial peptides termed Cn-AMPs have great potential to become new natural antibiotics [54].

### 3.6. Other Health Benefits

CW was also used to treat various ailments, including hepatic disorders, renal disorders, gastric disorders, and reproductive disorders. The use of TCW was recommended in cases of gastroenteritis and for urinary stone dissolution [55,56]. A recent anti-urolithiatic study involving male Wistar rats proved that CW prevented the adherence of crystals on renal tissues and reduced the number of crystals formed in the urine [38]. Other research suggests that CW increases the urinary excretion of potassium chloride, and citrate in humans, thus lowering the likelihood of stones [39]. In addition, it was reported that increasing concentrations of CW and fermented CW could decrease the number and size of struvite crystals that grew in a gel medium, indicating the antioxidant property and a marginal inhibitory effect during in vitro struvite crystallization, but anti-uropathogenic effects of CW were not found; meanwhile, fermented CW showed potential antioxidant, anti-uropathogenic, and anti-struvite urolithiatic properties [40].

TCW was also proven to have protective efficacy on heat stress-induced testicular damage in a murine system of male Wistar rats. The results indicated that TCW treatment could restore excess generation of oxygen radicals following the suppression of antioxidant capacity and augmentation of lipid peroxidation in murine testicles. The intervention also mitigated HS-induced inflammation via Nrf2 pathway activation, subsequently ameliorating testicular damage [41]. Furthermore, concentrated CW and its active constituent, shikimic acid, mitigated the oxidative damage induced by H_2_O_2_ in freshly isolated murine hepatocytes. This cytoprotection was mediated through coordinated regulation of three critical pathways: NF-κB inhibition (anti-inflammatory), PI3K/Akt/Nrf2 activation (antioxidant), and SAPK/JNK/Bax modulation (anti-apoptotic) [29].

Previous studies have reported that the oral administration of young coconut juice to ovariectomized rats can accelerate wound healing, which is associated with a significantly higher density of immunostaining for ER-α an ER-β in keratinocytes, fibroblasts, white blood cells, fat cells, sebaceous gland, skeletal muscles, and hair shafts and follicles. These findings demonstrated the significant wound-healing potential of young coconut juice, suggesting its therapeutic value for cutaneous repair [42]. In addition, studies have determined the biochemical properties of CW during the sprouting process, indicating that CW can be used as an adjuvant therapeutic food for children with mineral deficiencies.

## 4. Novel Processing Technologies for Coconut Water

Conventional thermal processing methods, such as pasteurization and sterilization, have been widely used to produce CW products by major commercial manufacturers like Coco-Cola, Pepsi CO, and Vita Coco [4]. Nevertheless, these methods can lead to the inactivation of heat-sensitive nutrients, degradation of volatile flavor compounds, and formation of undesirable chemical derivatives, which can make the CW less appealing to consumers [57,58]. In recent decades, there has been a growing interest in the development of innovative food processing technologies, including high-hydrostatic-pressure processing, high-pressure carbon dioxide, cold plasma, pulsed electric field, pulsed light, UV-C irradiation, ultrasound, ozone, and microfiltration, aimed at producing food products with superior preservation of nutritional and organoleptic properties. In this section, we systemically review the studies on the applications of these technologies to CW, focusing specifically on quality preservation, microbial reduction, and enzyme inactivation (Table 2).

### 4.1. High-Hydrostatic-Pressure (HHP) Processing

HHP processing applies pressures ranging from 100 to 800 MPa at moderate or elevated temperatures, typically employing water as the pressure transfer medium, in order to apply pressure instantaneously and uniformly throughout the food system [83]. It has been proven effective in reducing microbial counts with minimal effects on quality attributes and is now commercially applied to various food products, such as juices and beverages, meat products, and fruit and vegetable preparations [84,85].

Unfortunately, HHP is not considered a validated process for eliminating the spores of *Clostridium botulinum* in low-acid products (pH > 4.6), including CW with a pH range between 4.8 and 5.7 [59]. After HHP treatment at 550 MPa and 10 °C for 3 min, the total counts of inoculated non-toxigenic *C. botulinum* spores in CW were not significantly reduced and remained constant during the 61 days of storage at 4 °C, regardless of the initial dissolved oxygen content [59]. Storage of HHP-treated CW at 10 and 20 °C also showed no growth of *C. botulinum*, but there was a significant increase in total aerobic counts within 10 and 4 days, respectively, suggesting the limited capacity of HHP to inactivate all spoilage microorganisms. By supplementing CW with selected germinants, amino acids, and nutrient-rich laboratory media (TPGY broth), the authors concluded that CW was deficient in some essential nutrients required by *C. botulinum* to grow.

Raghubeer et al. [4] processed CW at 593 MPa and 4 °C for 3 min and also detected no growth of *C. botulinum* or toxin production during storage at 4 and 10 °C for 45 days. However, this absence of growth and toxin production was also noted in untreated samples, indicating the presence of naturally occurring inhibitory compounds in CW. Despite this observation, HHP was demonstrated to be effective in eliminating inoculated strains of *E. coli* O157:H7, *Salmonella*, and *Listeria monocytogenes* to <1 CFU/mL and producing a microbiologically stable (<2 log) product during storage at 4 °C for 120 days without the development of off odors and while maintaining a taste similar to fresh CW. However, given the concerns about the presence of *C. botulinum* in HHP-treated CW, further research is needed to explore hurdle approaches to enhancing the inactivation efficiency of *C. botulinum* and fully accomplish the potential of HHP technology for CW products.

### 4.2. High-Pressure Carbon Dioxide (HPCD)

HPCD technology utilizes sub- or supercritical carbon dioxide (CO_2_, T_c_ = 31.1 °C, P_c_ = 7.38 MPa), which exhibits properties that combine the low viscosity of a gas, intermediate diffusivity, and the high density of a liquid. These unique characteristics have led to the development of HPCD for a wide range of food processing applications, including preservation, extraction, encapsulation, and drying [86]. Furthermore, CO_2_ offers several advantageous properties. This Generally Recognized as Safe (GRAS)-certified material is non-toxic, cost-efficient, widely accessible, and easily removable.

To achieve the pasteurization of CW using HPCD, Cappelletti et al. [61] optimized the processing conditions, including the pressure, temperature, and treatment time. The optimized HPCD treatment, conducted at 12 MPa and 40 °C for 30 min, proved effective in achieving a 5-log (CFU/mL) reduction in mesophilic microorganisms, lactic acid bacteria, yeasts, and molds, as well as a 7-log (CFU/mL) reduction in total coliforms. Moreover, despite HPCD causing a decrease in pH and volatile fractions of CW, it retained nutritional and volatile compounds, as well as sensory attributes, significantly better than thermal treatment at 90 °C for 1 min. A detailed analysis of the volatile compound composition revealed that HPCD treatment reduced short- and medium-chain alcohols. In contrast, thermal treatment led to an increase in oxidated compounds, such as aldehydes and 2-acetyl-1-pyrroline, characterized by low odor thresholds with aromas of “cooked rice” and “popcorn” [62]. Additionally, sensory discrimination analysis showed no significant difference between HPCD-treated and untreated CW. However, when CW was treated solely with HPCD under the aforementioned optimized conditions, it exhibited microbiological instability after only 7 days of storage at 4 °C [63]. In contrast, combining HPCD with ultrasound at 12 MPa, 40 °C, and 10 W for 15 min extended the shelf life to 4 weeks [63].

### 4.3. Cold Plasma (CP)

Plasma, considered the fourth state of matter alongside solid, liquid, and gas, is generated when a single or combination of gases is excited by a high electric field strength using various electric discharge methods [87]. Among these methods, dielectric barrier discharge (DBD) is the most commonly employed. Plasma primarily consists of partially or fully ionized gases, encompassing the coexistence of positively and negatively charged ions, free radicals, excited molecules, UV photons, and other reactive species [88]. CP is generated at ambient temperature and atmospheric pressure and has been extensively utilized for food preservation. It enables the inactivation of a range of foodborne pathogenic and spoilage microorganisms and enzymes without significantly compromising the nutritional and organoleptic properties of foods [87].

Several studies have demonstrated that CP treatments have minimal or negligible effects on several physiochemical properties of CW, such as pH, total titratable acid (TTA), total soluble acid, ascorbic acid, and color parameters [64,65,66,67]. Nevertheless, the magnitude of these effects is highly dependent on the treatment conditions, including voltage, treatment time, and gas composition. For instance, Chutia and Mahanta [64] observed a significant reduction in the total phenolic content of CW when the CP treatment time was increased to 2 min, with a more pronounced decrease at higher voltages. Mahnot, Mahanta, Farkas, Keener, and Misra [67] demonstrated that processing CW with cold plasma generated in air did not affect the pH but reduced the TTA, whereas CP generated in M65 (65% O_2_, 30% CO_2_, 5%N2) lowered the pH without affecting the TTA.

When considering microbial safety, CP has been shown to achieve a reduction of approximately 1–2 log (CFU/mL) in natural or inoculated microbial load [66,67,68]. However, this level of inactivation is insufficient to ensure a satisfactory shelf life for CW under refrigerated conditions [64,67]. To enhance the microbial inactivation efficiency of CP on CW, the addition of citric acid has been investigated. A minimum reduction of 5 log (CFU/mL) in inoculated *E. coli*, *L. monocytogenes*, and *Salmonella*, as well as a shelf life of 48 days at 5 °C, was achieved with the addition of 400 ppm of citric acid to CW prior to CP treatment [66,67]. Additionally, Chutia and Mahanta [64] demonstrated that a blended beverage comprising CP-treated CW and orange juice, with the addition of ascorbic acid, maintained a shelf life of 35 days when stored at 6 °C in a glass bottle.

CP processing has also been demonstrated to achieve an inactivation rate of above 70% for POD in CW, with the effectiveness being influenced by processing parameters, such as frequency, voltage, and treatment time [64,65]. Moreover, it was found that compared to PPO, POD has stronger resistance to CP; when aiming for half of the maximum activity value, the processing time required for PPO is always shorter than that for POD, at 18 kV, 23 kV, and 28 kV [68].

### 4.4. Pulsed Electric Field (PEF)

PEF technology operates by applying intermittent high-voltage direct-current pulses, typically ranging from 100 to 300 V/cm up to 20–80 kV/cm, for very short durations (microseconds to milliseconds) [89,90]. These pulses pass through a food product positioned between two electrodes. When the electrical pulses reach a sufficient intensity, they can induce electroporation and potentially lead to dielectric breakdown [88]. This can cause leakage of intracellular compounds towards the external environment, ultimately resulting in cellular rupture [88]. Numerous studies have demonstrated the potential of PEF in various food processing applications, such as cold pasteurization, extraction of bioactive compounds, as well as drying, dehydration, and freezing processes [90].

Despite extensive research on the application of PEF technology for cold pasteurization of various juices, there is limited literature on its application to CW. Tongdonyod et al. [69] demonstrated the feasibility of combining PEF with mild heat treatment to achieve microbial inactivation while preserving the fresh-like characteristics of CW. The optimal PEF conditions, at 22.5 kV/cm, 119 kJ/L, and 40 °C, were found to inactivate the counts of *E. coli* K12 and *Listeria innocua* counts by 6.6 and 5.9 log (CFU/mL), respectively, and extend the shelf life to over 35 days at around 8 °C, comparable to thermal pasteurization at 85 °C for 10 min. Furthermore, PEF treatment resulted in fewer changes in physiochemical properties, sensorial qualities, and volatile flavor profiles of CW compared to thermal pasteurization. However, PEF treatment increased the PPO activity of CW by 17%, while the POD activity decreased by 78%.

### 4.5. Pulsed Light (PL)

PL relies on the application of a series of high-intensity light pulses ranging from 0.01 to 50 J/cm^2^ and short durations, typically between 1 μs and 0.1 s [91]. These pulses encompass an intense, broad spectrum spanning from 200 to 1100 nm, which includes ultraviolet (UV) light, visible light (VL), and infrared (IR). The lethal effect of PL on microorganisms is generally attributed to photo-chemical, photo-thermal, and photo-physical mechanisms. Among these, the photo-chemical effect plays a significant role, wherein light photons of UV-C wavelengths, notably 253.7 nm, are absorbed by microbial DNA, thus leading to cell death [91].

By optimizing pulsed light (PL) processing conditions for CW, including the PL fluence rate, exposure time, input voltage to the lamp, and distance between the lamp and the sample, a significant reduction of 5.33 log (CFU/mL) in inoculated *E. coli* was achieved. Additionally, there were reductions of 5.54 log (CFU/mL) and 4.67 log (CFU/mL) in the aerobic plate count and yeast and mold populations, respectively [70,72]. Furthermore, Basak et al. [71] demonstrated that PL treatment at 2.5 kV for 2.5 min (1073 J⋅cm^2^) ensured at least a 5-log (CFU/mL) reduction in *E. coli*, *Bacillus cereus*, and *L. monocytogenes*. Nevertheless, complete inactivation of POD and PPO enzymes required more intense PL treatment, at 2.9 kV for 5 min (1073 J/cm^2^) and 2.9 kV for 6 min (3586 J/cm^2^), respectively. The milder PL conditions for microbial safety of CW had no adverse effects on its physiochemical properties and sensory attributes. In contrast, more intense PL conditions aimed at achieving both microbial safety and enzymatic stability caused some damage, although they still resulted in higher total phenolics, ascorbic acid, and sensory scores compared to thermal processing at 90 °C for 3 min.

### 4.6. UV (UV-C) Irradiation

Ultraviolet (UV) light, which spans the electromagnetic spectrum from 100 and 400 nm, has been approved by the United States Food and Drug Administration (FDA) as an alternative technology for pasteurizing fruits and vegetable juices [75]. The germicidal effectiveness of UV light stems from its ability to initiate a complex series of chain reactions within cellular structures, leading to DNA damage [88]. As mentioned earlier, the highest microbial inactivation efficiency is achieved with light in the UV-C region (200–280 nm), as the absorbance of DNA at 253.7 nm closely matches the emitted wavelength.

Several studies have examined the efficiency of UV-C irradiation at 254 nm for nonthermal pasteurization of CW. Bhullar et al. [74] reported that UV-C treatment at 30 mJ/cm^2^ inactivated *E. coli*, *Salmonella Typhimurium*, and *L. monocytogenes* by above 5 log (CFU/mL) and bacteriophage T1UV by 4.73 log (CFU/mL). However, bacteriophage MS2 exhibited greater resistance to UV-C, requiring approximately 120 mJ/cm^2^ to achieve nearly 5-log inactivation. Notably, UV-C irradiation ranging from 100 to 400 mJ/cm^2^ did not produce cytotoxic effects on normal human intestinal cells or normal mouse liver cells. In terms of enzyme activity, UV-C treatment at 400 mJ/cm^2^ reduced the PPO and POD activity by 94 and 93%, respectively, without significant loss of essential amino acids, although there were slight changes in sensory attributes [18]. In contrast, Augusto et al. [77] discovered that PPO exhibited higher resistance compared to POD to UV treatment of CW within the wavelength range between 250 and 740 nm, with a peak between 400 and 420 nm. Specifically, PPO and POD activity decreased by 98 and 99%, respectively, after 30 min of UV processing. Additionally, Gabriel [76] discovered that common physicochemical stresses, such as acidity, desiccation, or their combinations, increased the exposure times and UV-C energy dose values required to achieve a 90% reduction in the population of inoculated cocktails of *E. coli* O157:H7, *S. enterica*, and *L. monocytogenes* in coconut liquid endosperm beverages, indicating increased resistance to UV-C treatment.

Maguluri et al. [73] investigated the potential of UV-C irradiation at 280 nm for the inactivation and photoreactivation of common food pathogens in CW. They found out that the UV-C doses required to achieve a 5-log reduction in the counts of *E. coli* and *L. monocytogenes* were 71 and 20.3 mJ/cm^2^, respectively. This study also demonstrated that UV treatment at 71 mJ/cm^2^ resulted in minor changes in the color, ascorbic acid concentration, and some sensory attributes of CW, while having no impact on other physiochemical properties, including pH, TTA, Brix, total phenolic content, mineral content, and sugars.

### 4.7. Ultrasound

Ultrasound is defined as a sound wave that exceeds the audible frequency range (20 kHz). It operates by inducing acoustic cavitation, which occurs when larger bubbles are generated, undergo expansion, and subsequently undergo rapid collapse, resulting in the release of a significant amount of energy [88]. Ultrasound possesses numerous advantages, including its eco-friendliness, cost-effectiveness, and non-destructiveness, and has been applied in various food processing techniques, such as filtration, freezing and crystallization, drying, sterilization/pasteurization, and extraction [92]. However, using ultrasound alone for food treatment has limited efficiency in achieving enzyme and microbial inactivation [78,79]. For instance, while ultrasound treatment at 60% amplitude with a 6 s pulse at a 20 kHz frequency for 6 min retained the initial quality parameters of CW, spoilage occurred within one week under both ambient and refrigerated conditions [17]. The addition of nisin at a final concentration of 5000 IU/200 mL to ultrasound-treated CW extended its shelf life to two weeks under refrigerated conditions but significantly decreased its nutritional and nutraceutical contents [17].

Combining ultrasound with other processing technologies, particularly thermal treatments, has demonstrated promise and potential in enhancing microbiological safety while maintaining the quality of food products [93]. POD has higher resistance to both ultrasound and thermal treatments, with its activity decreasing by 27 and 50% after ultrasound treatments at 286 W/L and 20 kHz for 30 min and thermal treatments at 80 °C for >30 min, respectively [78]. Pretreating CW with ultrasound has been demonstrated to sensitize POD before thermal treatments, resulting in more uniform heat resistance [78]. Ribeiro, Valdramidis, Nunes, and de Souza [79] also showed the significant additive effect of ultrasound on thermal treatment in inactivating both POD and PPO. Total inactivation of PPO and POD can be achieved after ultrasound treatment with energies of 629.90 (T_max_ > 60 °C) and 655.80 (T_max_ > 60 °C) W/L, respectively. However, due to the potential generation of off-flavors caused by oxidation reactions, decomposition of bioactive compounds, and negative impacts on physiochemical properties (e.g., pH, color, texture) induced by ultrasound, further investigation is required to evaluate the quality attributes of CW, as well as its microbial safety.

### 4.8. Ozone

Ozone is a triatomic allotrope of oxygen known for its high oxidizing power, which can effectively inactivate a wide range of microorganisms such as bacteria, viruses, algae, and fungi [65]. Due to its ability to spontaneously decompose into oxygen without leaving hazardous residues on foods, ozone is considered a GRAS chemical. It has been approved by the FDA as an antimicrobial agent for direct application to food since 2001 [88]. The antimicrobial mechanism of ozone involves its interaction with polyunsaturated fatty acids (PUFAs) in the cell envelope, converting them into acid peroxides. Additionally, ozone oxidizes amino acids, peptides, proteins, and enzymes by attacking sulfhydryl groups within these molecules [88].

Ozone treatment, utilizing concentrations within the range of 0.075 to 0.37 mg/mL and achieving over 50% absorption by CW, effectively decreased the activity of POD to undetectable levels, without altering its physicochemical properties or chemical compositions [65]. Nevertheless, Rajashri, Roopa, Negi, and Rastogi [17] demonstrated that ozone processing at 0.02 mg/mL led to spoilage of CW within one week of refrigerated storage. On the other hand, the combination of nisin and ozone processing extended the shelf life of CW to three weeks and inactivated both POD and PPO activity to undetectable levels while preserving its physicochemical, nutritional, and nutraceutical properties.

### 4.9. Microfiltration

Microfiltration (MF) is a process that ideally only rejects suspended solids while allowing proteins to pass freely through the membrane [94]. The operational principle of MF relies on physical size exclusion, where the membrane’s pore structure acts as a selective barrier based on particle dimensions [8]. This process has the potential to achieve pasteurization and clarification simultaneously. The combination of MF and additives has shown promise in preserving CW while maintaining its physiochemical properties. Purkayastha et al. [80] used a two-stage filtration system, including membrane filters with pore sizes of 0.8 and 0.45 μm, to process CW, followed by the addition of L-ascorbic acid. During storage at 4 °C for 21 days, methyl-α-D-rhamnopyranoside (the primary glycoside detected in micro-filtered CW) was retained, while the formation of free fatty acids was controlled, and rancidity was delayed. The same research group also investigated the preservation of CW using MF with additional additives, including citric acid (200 mg/L), ascorbic acid (180 mg/L), and L-cysteine (90 mg/L), using glass and plastic bottle packaging [81]. The results showed that both package methods could store CW at 4 °C for 46 days with acceptable sensory results, while CW remained sterile for 180 days in glass bottles. Additionally, MF followed by the addition of citric acid (200 mg/L), ascorbic acid (180 mg/L), and orange honey (5%, *w*/*v*) and packaging in glass bottles with headspace flushed with nitrogen achieved sterility of CW at 4 °C for 190 days, while maintaining its sensory attributes for 90 days [82].

## 5. Conclusions

CW has emerged as a popular functional beverage due to its rich nutritional profile, such as sugars, minerals, and vitamins, as well as its diverse biological activities, including antioxidant anti-inflammatory, anti-cancer, cardioprotective, and antimicrobial effects. To develop CW products with superior nutritional and organoleptic properties, innovative food processing technologies, such as high-hydrostatic-pressure processing, high-pressure carbon dioxide, cold plasma, pulsed electric field, pulsed light, UV-C irradiation, ultrasound, ozone, and microfiltration, have been explored. These methods offer promising alternatives to traditional thermal processing by preserving CW’s quality attributes while achieving microbial and enzyme inactivation.

Coconut is a vital agricultural commodity in tropical regions, contributing significantly to global trade and economic growth. However, in some areas, the utilization of coconut resources remains underdeveloped, with mature CW often discarded, leading to environmental pollution, resource wastage, and challenges for sustainable industry growth. To address these challenges, efforts should focus on enhancing the technological content of coconut products, expanding market opportunities, and improving coconut meat processing capabilities. Additionally, identifying natural enzyme inhibitors to prevent enzymatic degradation in CW offers a promising research direction for better maintaining its nutritional value, taste, and flavor. Further exploration of coconut protein’s nutritional and health benefits could support the development of functional food products, advancing the food processing industry and contributing to the global tropical economy. By-products from the coconut industry also hold significant potential as nutraceuticals for managing metabolic disorders, offering both health and economic benefits on a global scale. 

## Figures and Tables

**Table 1:** Bioactivities of coconut water and potential mechanisms.

Bioactivity	In Vivo/In Vitro Model	Treatments	Results	Potential Mechanisms	Reference
Antioxidant	Wistar albino rats	Orally given 10 mL, 20 mL, 30 mL, and 40 mL of the coconut water extract for 28 days	Increased MDA, SOD, GSH, and vitamin C	/	[34]
	Murine hepatocytes	CWC (200 and 400 μg/mL)	Mitigated the oxidative damage induced by H_2_O_2_	Inhibition of NF-κB, activation of PI3K/Akt/Nrf2, reduction in apoptosis by the SAPK/JNK/Bax pathway	[29]
Anti-inflammatory	Rat paw edema model	4 mL/100 g dose oral treatment	Inhibition of 42.52% for young CW and 25.94% for mature CW	Inhibition of Nos2 mRNA and iNOS protein expression through AKT and JNK signaling pathways	[14]
	Hepatocytes isolated from male Sprague–Dawley rats	TCW (*v*/*v*) and sterile water mixed to 2 X William E	Improved hepatocyte viability and protected hepatocytes against cytokine-mediated cell death	Suppressed IL-1β-mediated increase in Nos2, Tnf, and Il6 mRNA and increased heme oxygenase 1 (HMOX1) protein Inhibited iNOS expression through activation of AKT and JNK pathways	[35]
Anti-proliferative	Human glioma cell lines 1321N1 and U87MG	Incubation with concentrations from 0.25 to 2.0 mM for 72 h	IC_50_ values of 1.25 and 1.85 mM	The long hydrophobic C-terminal sequence penetrates the membrane core region, driving the translocation of Cn-AMP2 across the cancer cell membrane to attack intracellular targets and induce anti-proliferative mechanisms	[15]
Cardioprotective effects	Male Sprague–Dawley rats	4 mL/100 g of body weight	Lowered the systolic blood pressure, reduced serum triglycerides and free fatty acids	Inhibition of lipid peroxidation, upregulation of antioxidant status, and improved insulin sensitivity	[11]
	Male Sprague–Dawley rats	Oral administration of mature CW (4 mL/100 g body weight) for 45 days	Significant reduction in blood glucose and glycated hemoglobin levels, increased plasma insulin levels	Reduction in pancreatic damage induced by alloxan Stimulated cell regeneration	[32]
	Male albino rats (Sprague–Dawley)	Oral administration with TCW (4 mL/100 g/day for 30 days) and single-dose (250 mg/100 g) intravenous post-treatment with lyophilized TCW	Reduced oxidative stress and exerted antithrombotic effects	Scavenging free radicals; inhibition of platelet aggregation; and adhesion by inhibiting fibrinogen and enhancing the release of anti-aggregatory nitric oxide	[36]
	Male Sprague–Dawley rats	Oral MCW treatment (4 mL/100 g body weight) for 45 days	Reduce the concentration of blood glucose and HbA_1c_; increased contents of nitric oxide synthase, liver and plasma arginine, and urinary nitrite	Mediated by *L*-arginine-nitric oxide pathway	[13]
Antimicrobial	Apparently healthy Wistar (AHW) rats	Daily orogastric administration of various volumes of CW (0.5–2.0 mL) for four weeks	Inhibitory effects on the growth of *Escherichia coli*, *Proteus mirabilis*, *Pseudomonas aeruginosa,* and so on	/	[37]
Anti-urolithiatic	Male Wistar rats	/	Prevented the adherence of crystals on renal tissues and reduced the number of crystals formed in the urine	/	[38]
	Adult volunteers with no prior history of nephrolithiasis	Consumed 1.92 L of either Taste of Nirvana pure coconut water or tap water daily for four days	Significantly increased urinary citrate (29%), urinary potassium (130%), and urinary chloride (37%), without affecting urine pH, lowering the likelihood of stones	/	[39]
			Decreased the number and size of struvite crystals in the gel medium		[40]
Protection on murine system	Male Wistar rats		Protective effects on heat stress-induced testicular damage	Reversed the HS-induced proinflammatory state through activation of the Nrf2-assisted antioxidant response	[41]
Cutaneous wound healing	Ovariectomized rats	Oral administration of young coconut juice	Beneficial effects on cutaneous wound healing	Significantly increased density of immunostaining for ER-α and ER-β in keratinocytes, fibroblasts, white blood cells, fat cells, and so on	[42]

**Table 2:** Effects of nonthermal processing technologies on the quality attributes, microbial inactivation, and enzyme inactivation of coconut water.

Processing Technology	Treatment Conditions	Quality Attributes	Microbial Inactivation	Enzyme Inactivation	Reference
High hydrostatic pressure (HHP)	Pressure: 550 MPa; Time: 3 min; Temperature: 10 °C (Initial)	-	The counts of inoculated Type E *C. botulinum* and Group II *Clostridium* sp. were not significantly reduced and remained constant regardless of the initial dissolved oxygen content or storage temperature (4 and 20 °C).	-	[59]
	Pressure: 593 MPa; Time: 3 min; Temperature: 4 °C	-	The counts of inoculated *E. coli O157:H7*, *Salmonella*, and *L. monocytogenes* were reduced to <1 CFU/mL during storage at 4 °C for 54 and 75 days for Florida CW and Brazil CW, respectively. Additionally, the uninoculated CW sample was microbiologically stable (<2 log CFU/mL) during storage at 4 °C for 120 days.	-	[4]
	Pressure: 500 MPa; Time: 5 min; Temperature: Room temperature	The amino acids, total protein, sugars, phenols, ascorbic acid, and antioxidant capacity were retained after storage at 4 °C for 25 days. The color, aroma, flavor, and overall acceptability were retained.	The counts of total aerobic bacteria (TAB) and molds and yeasts (M&Y) were reduced to undetectable levels and remained below 2 and 1.3 log (CFU/mL), respectively, during storage at 4 °C for 25 days.	-	[60]
High-pressure carbon dioxide (HPCD)	Pressure: 8 and 12 MPa; Time: 5–60 min; Temperature: 22, 30, 35, 40, and 45 °C	The dry matter, soluble solids, sugars, and vitamin content were retained while the pH and volatile fraction were reduced. The sensory attributes were not affected.	12 MP, 40 °C, and 30 min were the optimal HPCD conditions to induce 5-log (CFU/mL) reductions in mesophilic microorganisms, lactic acid bacteria, yeasts, and molds and a 7-log reduction in total coliforms.	-	[61]
	Pressure: 12 MPa; Time: 30 min; Temperature: 40 °C	The short- and medium-chain alcohols were depleted and the sensory attributes were not affected.	-	-	[62]
	Pressure: 12 MPa; Time: 1–60 min; Temperature: 25, 30, 35, and 40 °C	-	2-log (CFU/mL) reductions in inoculated *S. enterica* were achieved when HPCD was conducted at 40 °C. For natural microbial flora, HPCD at 12 MP, 40 °C, and 30 min assured sufficient microbial inactivation, but the product was microbiologically unstable during storage at 4 °C. Combining HPCD with ultrasound (12 MPa, 40 °C, 15 min, and 10 W) assured a shelf life at 4 °C for 4 weeks.	-	[63]
Cold plasma	Voltage: 18 to 28 kV; Gap of dielectric barriers: 1.5 cm; Working gas: air; Time: 1–3 min	The total fatty acid was increased while the ascorbic acid, DPPH free radical scavenging activity, and transmittance were decreased. The total phenol content was decreased until the treatment time reached 3 min.	Around a 2-log (CFU/mL) reduction in microbial load was achieved.	Treatment at 18 kV and 2.85 min resulted in a residual POD activity of 22%.	[64]
	Frequency: 200, 400, 550, and 730 Hz; Voltage: 15 and 20 kV; Gap of dielectric barriers: 1.5 cm; Working gas: air; Time: 15 min	The physicochemical properties (pH, total soluble acids, titratable acidity, and color) were not significantly affected.	-	Increase in frequency promoted the inactivation of POD activity, with the smallest residual activity of 28% at 730 Hz.	[65]
	Voltage: 90 kV; Gap of dielectric barriers: 5 cm; Working gas: dry air; Time: 0.5–2 min	The pH, total titratable acid (TTA), and L* value were significantly decreased, and the conductivity, total soluble solids (TSS), ascorbic acid, transmission, and a* value were not affected.	At a plasma treatment time of 2 min, a 1.3-log (CFU/mL) reduction in inoculated *S. enterica* was achieved.	-	[66]
	Voltage: 90 kV; Gap of dielectric barriers: 5 cm; Working gas: dry air (78% N_2_, 21% O_2_ and traces of other gases) and modified air M65 (65% O_2_, 30% CO_2_, 5% N_2_); Time: 2 min	The pH was not affected but decreased using air and M65 gas, respectively. The total titratable acid (TTA) was decreased but not affected using air and M65 gas, respectively. The total soluble solids (TSS) was not significantly affected. The L* and b* values were not greatly impacted while the a* values were decreased.	For *E. coli*, 1.09- and 1.79-log (CFU/mL) reductions were achieved using air and M65 gas; while for *L. monocytogenes*, a 2.03-log (CFU/mL) reduction was achieved using air, and no microbial recoveries were obtained with M65 air by 24 h post refrigerated storage.	-	[67]
	Voltage: 18, 23, and 28 kV; Gap of dielectric barriers: 1.5 cm; Working gas: air; Time: 1–5 min	-	-	The times required for half-maximal activity values for POD were 0.84, 1.67, and 2.53 min at 18 kV, 23 kV, and 28 kV, respectively, and for PPO were 0.67, 1.18, and 1.35 min, respectively. POP was more resistant to cold plasma than PPO.	[68]
Pulsed electric field (PEF)	Electric field strength: 20–30 kV/cm; Specific energy: 80–120 kJ/L; Pulse width: 4 µs; Flow rate: 100 L/h; Pulse frequency: 130–350 Hz; Temperature: 30–40 °C	PEF treatment decreased the scores for sensory attributes and changed the volatile flavor profiles.	The optimized PEF treatment combining 40 °C with a field strength of 22.5 kV/cm and specific energy of 119 kJ/L decreased the count of inoculated *E. coli* K12 and *Listeria innocua* by 6.60 and 5.90 (CFU/mL), respectively.	PEF treatment increased the PPO activity by 17%, while it decreased the POD activity by 78%.	[69]
Pulsed light (PL)	Fluence rate: 8.1–756 J/cm^2^; Time: 0–0.25 min	-	PL treatment at 95.2 J/cm^2^ decreased the count of inoculated *E. coli* by 5.33-log (CFU/mL).	-	[70]
	Fluence rate: 127–3586 J/cm^2^; Time: 0–6 min	-	PL treatment at 465 J/cm^2^ decreased the counts of inoculated *E. coli*, *Bacillus cereus*, and *L. monocytogenes* by 5.12, 2.97, and 3.40 log (CFU/mL), respectively.	PL treatment at 2988 J/cm^2^ inactivated PPO and POD to <1% residual activity. PPO was more resistant to PL than POD.	[71]
	Fluence rate: 12.6–756 J/cm^2^; Time: 0.25–0.75 min	-	The optimized PL treatment with an input voltage of 1492 V, distance of 7.6 cm, and treatment time of 43 s (12 passes) decreased the aerobic plate count (APC) and yeast and mold (Y&M) by 5 and 4 log (CFU/mL), respectively.	-	[72]
UV (UV-C) irradiation	UV-C light wavelength: 254 nm; Reduction equivalent fluence (REF): 0–400 mJ/cm^2^	No significant loss in nine essential amino acids and sensory attributes were observed after UV-C treatment at 400 and 200 mJ/cm^2^, respectively.	-	UV-C treatment at 400 mJ/cm^2^ decreased the PPO and POD activity by 94 and 93%, respectively. PPO was more resistant to UV-C than POD.	[18]
	UV-C light wavelength: 280 nm; Reduction equivalent fluence (REF): 0–70 mJ/cm^2^	UV treatment did not affect the pH, titratable acidity, Brix, total phenolic content, mineral content, and sugars, but brought slight changes in the color measurement and ascorbic acid concentration. The sensory attributes were slightly decreased.	UV-C treatment at 71 and 20.3 mJ/cm^2^ decreased the counts of inoculated *E. coli* and *L. monocytogenes* by ≥5 log (CFU/mL), respectively.	-	[73]
	UV-C light wavelength: 254 nm; Reduction equivalent fluence (REF): 0–400 mJ/cm^2^	-	UV-C treatment at 30 mJ/cm^2^ can decrease the counts of inoculated *E. coli*, *S. Typhimurium*, and *L. monocytogenes* by ≥5 log (CFU/mL). UV-C treatment at 30 and 120 mJ/cm^2^ decreased the contents of T1UV-C and MS2 surrogate viruses by ≥5 log (CFU/mL), respectively.	-	[74]
	UV-C light wavelength: 254 nm; UV dose: 802 and 425.46 mJ/mL in 1.6 and 3.2 mm reactor, respectively; Reynolds number: 198.8, 397.7, 596.4	The physicochemical properties (pH, soluble solids, and density) were not significantly affected.	UVC treatment at Re 596.4 and 1.6 mm reactor decreased the counts of *E. coli* and *L. monocytogenes* by 5.27 and 4.18 log (CFU/mL), respectively.	-	[75]
	UV-C light wavelength: 254 nm; UV-C dose: 2.6 mW/cm^2^; Time: 0–0.4 min	-	Exposures to different sublethal stresses (i.e., acidification, desiccation) increased the exposure times (D) and UV-C energy dose values (D_UVC) necessary to reduce 90% of the population of *E. coli*, *S. enterica*, and *L. monocytogenes*.	-	[76]
	Light wavelength: 250–740 nm (peak between 400 and 420 nm); Nominal power: 400 W; Time: 1–60 min; Temperature: 25 °C	-	-	Treatment for 15 min decreased the POD and PPO activity by ~5% and ~8%, respectively. Treatment for 30 min decreased the POD and PPO activity by ~1% and ~2%, respectively. PPO was more resistant than POD.	[77]
Ultrasound	Ultrasonic power: 60% amplitude with 6 s pulse; Frequency: 20 kHz; Time: 1–10 min; Temperature: 20 °C	Ultrasound treatment for 6 min retained the initial quality attributes.	The samples stored at room and refrigerated temperatures were spoiled within a week. The addition of nisin reduced the bacterial population by 2 log (CFU/mL) and the counts of yeast, molds, and *E. coli* to undetectable levels, and achieved a storage time of 2 weeks in a refrigerated condition.	Combining ultrasound treatment with nisin inactivated the PPO, POD, and PAL activity by 50, 30, and 35%, respectively.	[17]
	Acoustic energy: 286 W/L; Frequency: 20 kHz; Time: 0–30 min; Temperature: 25 °C	-	-	Ultrasonic treatment for 30 min decreased the POD activity by around 27%.	[78]
	Acoustic energy: 448–717 W/L; Frequency: 20 kHz; Time: 5–15 min; Temperature: 20–80 °C	-	-	Ultrasonic treatment with acoustic energy > 500–550 W/L is required to promote a significant decrease in PPO and POD activity. Acoustic treatment with energies of 629.90 and 655.80 W/L can achieve total inactivation of PPO and POD, respectively.	[79]
Ozone	Ozone loads: 0.075–0.37 mg/mL; Temperature: 10–30 °C	The physicochemical properties (pH, total soluble acids, titratable acidity, and color) were not significantly affected.	-	No detectable activity of POD was found after all ozone treatments.	[65]
	Ozone loads: 20 mg/L at a flow rate of 1 L/min; Time: 1–10 min; Temperature: 25 °C	Ozone treatment for 5 min retained the initial quality attributes.	The samples stored at room and refrigerated temperatures were spoiled within a week. The addition of nisin achieved a storage time of 3 weeks in a refrigerated condition.	Combining ozone treatment with nisin inactivated PPO and POD activity to undetectable levels.	[17]
Microfiltration	Filter: 0.8 and 0.45 μm pore size; Additive: *L*-ascorbic acid	The addition of L-ascorbic acid to micro-filtered coconut water retained methyl-α-D-rhamnopyranoside (prime glycoside detected in micro-filtered coconut water), controlled the formation of free fatty acids, and delayed rancidity during storage at 4 °C.	-	-	[80]
	Filter: 0.8 and 0.45 μm pore size; Additive: ascorbic acid, citric acid, and *L*-cysteine	The sensory qualities remained stable during storage at 4 °C for 46 days. The pH and total soluble solids did not change significantly, while total titratable acidity and simple sugars increased.	The microbial load of coconut water in plastic bottles and glass bottles remained sterile during storage at 4 °C for 7 and 180 days, respectively.	-	[81]
	Filter: 0.8 and 0.45 μm pore size; Additive: citric acid, ascorbic acid, and orange honey	The physicochemical properties (pH, titratable acidity, total soluble solids, total simple sugars, free fatty acid, total reducing sugars) were not significantly affected.	No microbial count was detected on the total plate count agar and coliform plates during storage at 4 °C for 190 days.	The PPO activity was reduced while the PO and SNI activity were not changed.	[82]

## Data Availability

No new data were created or analyzed in this study. Data sharing is not applicable to this article.

## Abbreviations

The following abbreviations are used in this manuscript:

USDA	United States Department of Agriculture
TSS	Total Soluble Solids
CW	Coconut Water
TCW	Tender Coconut Water
HHP	High Hydrostatic Pressure
HPCD	High-Pressure Carbon Dioxide
CP	Cold Plasma
PPO	Polyphenol Oxidase
POD	Peroxidase
PEF	Pulsed Electric Field
PL	Pulsed Light
UV	Ultraviolet
MF	Microfiltration
